# General Medicine and Therapeutics

**Published:** 1896-07

**Authors:** 


### SELECTIONS AND ABSTRACTS.

### GENERAL MEDICINE AND THERAPEUTICS.

### Cyclic Albuminuria.

Pierre Marie (*Sem. Med.*, February 5, 1896) reports a case and discusses the subject. He defines the disease as albuminuria, (1) occurring in apparently healthy subjects, (2) being intermittent in its course, and (3) subject to certain conditions. As regards (1), the discovery of albumen may be perfectly fortuitous, or the patient may suffer from general debility, heaviness of the limbs, palpitations, headaches and hemorrhages (epistaxis or hemoptysis). (2) Intermittence: many healthy persons may have transitory albuminuria under certain conditions. Hwass found albumen in 98 out of 635 apparently healthy soldiers (15.4 per cent.), the quantity of which was not influenced by exercise. Capitan and Spiegler found albumen, probably from reflex causes, always present in one hundred cases of scabies. Thus transitory must be distinguished from true cyclic albuminuria. (3) Conditions: it is doubtful whether cases which are influenced by food or cold are examples of this disease, for in perfectly typical cases they have often no effect. The cardinal symptom is that albumen is present only when the upright position is assumed, and vanishes on lying or sitting down, even when hard work is done by the patient on an exercise apparatus ("postural albuminuria" of A. W. Stirling). In the case reported by the author, however, albumen appeared sometimes when the patient was in bed, at times of great annoyance or stormy weather. *Albumen.*—The quantity is always small. Its chemical composition varies with the individual and in the same individual at different times. Serum albumen, peptone, propeptone, nucleo-albumen, etc., have been found. *Urine.*—The quantity is not much affected. If at any time it is less than normal there is said to be a corresponding increase in the albumen. The specific gravity varies, but is usually normal or higher (1020 to 1030). The acidity is usually

*plus*. Teissier gives the following cycle in this disease: (*a*) *plus* excretion of coloring matter, (*b*) albuminuria, (*c*) *plus* excretion of urates, (*d*) *plus* excretion of urea. *Etiology*.—(1) Sex, more common in females. (2) Age, adolescence and early adult life. (3) Heredity, especially in children of gouty and arthritic parents (Teissier). (4) Disputed antecedents are renal affections, colds, overwork, and infective fevers. After reviewing the various theories as to the nature of this complaint, the author states that he considers it to be a special morbid entity, probably depending on perverted function of sympathetic nerves, and having a close analogy to migraine. He does not agree with those who believe the cause to be a "minimal" organic renal lesion, as the almost indefinite time the disease can last without bad effects, and its not being influenced by the quality of the food, are against that theory. Casts are not generally present, but Marie does not consider the presence of casts in small numbers to indicate organic disease, for Hwass, by means of centrifugalization, found them sixty-nine times in seventy-four healthy soldiers. Of these, forty-eight alone had albuminuria, and that of a transitory character.—*Brit. Med. Jour.*, *June 6, '96.*

---

### PRECAUTIONS FOR CONSUMPTIVE PATIENTS.

The following precautions should be taken by patients with a tendency to consumption (adopted by the Bournemouth Medical Society):

The expectoration must be destroyed, and must not be allowed to get dry, as it may then become pulverized and diffused through the air. If inhaled, it may thus communicate the disease to other persons, or cause fresh attacks in the same invalid. On no account, therefore, must pocket handkerchiefs be used for expectoration. If used for wiping the mouth they should be put aside after use. A spitting-cup (or pocket spitting-flask for-out-of doors), containing a little disinfectant solution, should always be used. Small pieces of linen or calico, or Japanese paper handkerchiefs, should also be carried, which should be used only in case of absolute necessity, and burnt as soon as possible afterwards.

Spoons, forks, cups, and other articles of this kind should be thoroughly washed before being used by other persons.

The patient should always occupy a separate bed, and if a nurse is not required, a separate bedroom.

Fresh air and sunlight are most essential. The windows both of living rooms and bedrooms should be kept open as much as possible, although direct draughts should be carefully avoided. The chimney should never be blocked up.

With these precautions no danger of infection need be feared.

The following recommendations as to the cleansing of rooms occupied by consumptive patients were adopted by the Bournemouth Medical Society:

It is an established fact that consumption is sometimes caused by the breathing of matter coughed up by consumptive persons, which, when it becomes dry, may be blown about in the air as dust.

It is desirable, therefore, that rooms which have been constantly occupied by consumptive persons should be very thoroughly cleansed when vacated, so that a declaration or assurance to this effect may be given, if required, to the next occupants of such rooms.

The process of cleansing should include the thorough brushing and exposure in the open air of stuffed furniture; carpets, curtains, hangings, and bedding should be "cleaned," or thoroughly shaken and exposed in the open air for several hours, during sunshine if possible. Where facilities for this do not exist, these articles should be sent to the steam disinfector. Floors, paint, and furniture should be treated with some disinfectant, and then washed with soap and water. Walls should be rubbed down with new bread. Ceilings should be thoroughly dusted, and every conceivable nook for dust should be carefully cleaned. The room should be freely ventilated for at least twenty-four hours with wide open windows.

Rooms which are occasionally occupied by consumptives should be freely ventilated every day, and kept thoroughly clean; they should be thoroughly cleansed, as above described, at least twice a year.

The corporation have adequate arrangements for disinfections, etc. The regulations and tariff can be obtained at the office of the sanitary inspector.—*Sanitary Journal.*

